# Chemokine CXCL1 as a potential marker of disease activity in systemic lupus erythematosus

**DOI:** 10.1186/s12865-021-00469-x

**Published:** 2021-12-27

**Authors:** Yanli Zeng, Qiaoduan Lin, Liang Yu, Xuelian Wang, Yiqiang Lin, Yan Zhang, Shuidi Yan, Xinxin Lu, Yijing Li, Weibin Li, Yun Xiao

**Affiliations:** 1 Center of Clinical Laboratory, Zhongshan Hospital, School of Medicine, Xiamen University, Xiamen, 361004 China; 2 Ultrasonography Department, Zhongshan Hospital of Xiamen University, Xiamen, 361004 China; 3 Department of Obstetrics and Gynecology, Zhongshan Hospital of Xiamen University, Xiamen, 361004 China; 4 Institute for Laboratory Medicine, The 900Th Hospital of Joint Logistic Support Force, Fuzhou, 350025 China

**Keywords:** CXC ligand 1 (CXCL1), Growth-related oncogene-α (GRO-α), High-avidity IgG ANAs (HA IgG ANAs), Systemic lupus erythematosus (SLE), Lupus nephritis (LN)

## Abstract

**Objectives:**

The chemokine CXCL1, known as growth-related oncogene α (GRO-α), is a potent chemoattractant and regulator of neutrophils. The purpose of our study was to evaluate the regulatory response of CXCL1 in the serum of patients with systemic lupus erythematosus (SLE) in the active stage of disease and to assess whether it was implicated in the pathogenesis/inflammatory process in lupus.

**Methods:**

CXCL1 serum concentrations were examined in 90 SLE patients, 56 other autoimmune diseases (OADs) patients and 100 healthy controls using enzyme-linked immunosorbent methodology.

**Results:**

SLE patients exhibited significant increases in serum CXCL1 concentrations [1492.86 (735.47–2887.34) pg/ml] compared with OADs patients [155.88 (10.77–366.78) pg/ml] and healthy controls [13.58 (8.46–37.22) pg/ml] (*p* < 0.001). Moreover, the level of CXCL1 decreased as the level of anti-dsDNA IgG decreased after treatment between the anti-dsDNA-positive SLE patients and the anti-dsDNA-negative SLE patients. Additionly, serum CXCL1 concentrations were related to different disease activity levels in SLE and lupus nephritis (LN) and high avidity of IgG ANAs (HA IgG ANAs) (*p* < 0.05). Furthermore, CXCL1 serum concentrations were significantly correlated with the SLE Disease Activity Index(SLEDAI) score, relative avidity index (RAI) of HA IgG ANAs and the levels of anti-dsDNA IgG, CRP, ESR, albumin, C3 and C4.Additionally, Statistical analysis revealed that positivity for IgG ANA (*p* < 0.001), the presence of HA IgG ANAs (*p* = 0.001) and the logarithmic level of anti-dsDNA IgG (*p* = 0.021) were significantly associated with the logarithmic level of CXCL1 with standard partial regression coefficients (95% CI) of 2.371 (1.734–3.009), 1.231 (0.52–1.937) and 0.409 (0.062–0.755), respectively. Finally, using cutoff points of 1182.17 pg/mL and 1500.31 pg/mL, serum CXCL1 levels had a similar sensitivity of 76% and specificity of 100% and 75% for the diagnosis of active SLE and LN, respectively.

**Conclusions:**

Serum CXCL13 concentrations might represent a potential marker of disease activity in systemic lupus erythematosus.

## Introduction

Systemic lupus erythematosus (SLE) is a common autoimmune disease involving multiple organs and systems. SLE is characterized by the production of a large number of autoantibodies and the deposition of various immune complexes in target tissues [1]. Anti-dsDNA antibodies exhibit high specificity for SLE and are associated with disease activity [2]. However, in different studies, the prevalence of anti-dsDNA IgG has been found in approximately 27.8–50% of SLE patients despite clinically active symptoms [3–5]. The role of chemokines and cytokines in the pathogenesis of SLE and lupus nephritis (LN) has been widely accepted [6], and previous studies have demonstrated the therapeutic benefits of chemokine/chemokine receptor or cytokine/anti-cytokine autoantibody blockade in experimental SLE [7, 8].

CXC ligand 1 (CXCL1) was originally isolated and characterized based on its growth stimulatory activity against malignant melanoma cells [9]. CXCL1 is also known as growth-related oncogene (GRO) alpha and is a member of the CXC chemokine family with a three amino acid motif (ELR: glutamate, leucine, arginine), this chemokine plays an important role in inflammation, angiogenesis, tumorigenesis and wound healing [10]. CXCL1 is a potent neutrophil chemoattractant and activator that mainly acts through CXCR2 receptors. Although elevated CXCL1 expression has been reported in several human cancers, the role of CXCL1 and its receptor CXCR2 in autoimmune diseases is unclear. Kanapathippillai et al. demonstrated that in (NZBxNZW)F1 (B/W) mice with lupus nephritis, chemokines CXCL1, CXCL2, CXCL5, CCL2, CCL7, and CCL20 were increased in primary mesangial cells stimulated by either nucleosomes alone or nucleosome-IgG complexes accompanied by infiltration of neutrophils, macrophages, and T and B cells [11]. Additionally, Wang et al. [12] reported that in a murine model of lupus, pristane-treated wild-type (WT) mice exhibited elevated CXCL1, MCP-1, anti-snRNP and anti-dsDNA levels compared with untreated mice; however, CXCL1, MCP-1 and anti-snRNP levels were reduced in pristane-treated P-selectin glycoprotein ligand-1-deficient (Psgl-1^−/−^) mice compared to pristane-treated WT mice. These results verified that CXCL1 may be involved in the course of SLE, especially LN, and that CXCL1 is an important factor that regulates active leukocyte recruitment into inflamed tissue. Furthermore, in the case of lupus nephritis, the temporal course of the event is believed to place the production of chemokines at a very early moment in the disease process, which is consistent with their proposed role as an early mediator of renal inflammation.

The purpose of this study was to investigate whether serum levels of chemokine CXCL1 (1) are elevated in patients with active SLE, (2) are related to disease activity and influencing factors, and (3) are a potential diagnostic marker to distinguish active SLE from inactive SLE and LN from non-LN.

## Materials and methods

### Study participants

All patients were consecutively recruited from Zhongshan Hospital, Medical College of Xiamen University between April 2019 and September 2021. Ninety patients (7 males and 83 females, median age 33.5 years) with SLE were enrolled in this study. All patients with SLE were diagnosed according to the 2019 European League Against Rheumatism (EULAR)/American College of Rheumatology (ACR) classification criteria [13]. Disease activity in patients with SLE was assessed against the SLE Disease Activity Index 2000 (SLEDAI-2K) [14, 15], and the diagnosis of lupus nephritis (LN) was evaluated as previously described [1, 4, 16]. Fifty-six patients (12 males and 44 females, median age 37 years) were diagnosed with other autoimmune diseases (OADs) according to their diagnostic criteria. We recruited patients with rheumatoid arthritis (RA, 22 cases), Sjögren’s syndrome (SS, 19 cases), mixed connective tissue disease (MCTD, 12 cases), systemic sclerosis (SSC, 2 cases), and anti-phospholipid syndrome (APS, 1 case). In addition, we recruited 100 age- and sex-matched patients (13 males and 87 females, median age 33 years) without any risk factors or chronic diseases.

We excluded pregnant women and patients with various cancers and known immune system defects (such as HIV infection, hematologic diseases or transplantation history).

### Quantification of CXCL1 levels and other laboratory parameter testing

CXCL1 serum levels were analyzed using human Growth-Regulated Oncogenea/Melanoma Growth Stimulating Activity (GRO ɑ/MGSA) ELISA kit according to the manufacturer’s instructions (CUSABIO, Wuhan, China). Serum samples were added to a well and incubated for 2 h at 37 °C. After removing the liquid of each well without washing, 100 µL of biotin-conjugated antibody specific for GROɑ were added to each well and incubated for 1 h at 37 °C. After washing, avidin conjugated Horseradish Peroxidase (HRP) was added to the wells. Following a wash to remove any unbound avidin-enzyme reagent, tetramethyl-benzidine (TMB) was applied as a substrate and incubated for 30 min at 37 °C. The reaction was stopped with 2 N H_2_SO_4_ and determined the optical density of each well within 5 min using a microplate reader set to 450 nm.This kit detection rang was 31.25–2000 pg/ml. Moreover, no significant cross-reactivity of interference between human GROɑ and analogues was observed.

We also examined the serum concentration of anti-dsDNA IgG using ELISA based on the manufacturer’s protocol and our previous studies [1]. The optical density (OD) was measured at 450 nm using an ELISA Reader (Thermo Fisher Technology Co., Ltd, Shanghai, China). CXCL1 and anti-dsDNA IgG levels were quantified using standard curves. All samples were measured in duplicate. Peripheral blood samples were immediately centrifuged at 1000 g for 15 min, and serum was removed. Then, samples were stored at − 80 °C.

In the standard test, the total IgG ANAs and avidity of IgG ANAs were determined by indirect immunofluorescence (IIF) assays. The relative avidity index (RAI) was used to assess the avidity of IgG ANAs, as we previously reported. Serum levels of complement C3/C4, C-reactive protein (CRP) and albumin were determined by nephelometry using the Image Immunochemistry System (Roche Cobas 8000, Germany). In addition, the erythrocyte sedimentation ratio (ESR) (Succeeder Technology Inc., Beijing, China) and number of neutrophils (Sysmex Corporation, XN9000, Japan) were determined by instrument methods.

### Statistical analysis

The differences among the three study groups were evaluated using the nonparametric Mann–Whitney *U* test. Pearson’s chi-square test was used to calculate the differences between IgG ANAs and high avidity IgG ANAs. Spearman’s rank correlation was used to analyze the correlations between CXCL1 concentrations and disease activity parameters. To further verify laboratory parameters influencing CXCL1 levels, stepwise multiple linear regression analysis was performed. Receiver operator characteristic (ROC) curves were applied to analyze CXCL1 as a diagnostic marker to distinguish active SLE from inactive SLE and LN from non-LN. Data are presented as the median ± interquartile range (IQR) unless otherwise noted. All procedures were analyzed based on *p* < 0.05 as the level (two sided) of statistical significance. SPSS software and GraphPad Prism (version 20.0, IBM Inc., New York, USA; version 8.0.1, GraphPad Prism Software Inc. San Diego, CA, USA) were used for all statistical analyses.

## Results

### Study participant characteristics

Table 1 lists the characteristics of the participants in this study. In the study, no significant differences in sex (χ^2^ = 5.685, *p* = 0.058) or age (χ^2^ = 5.286, *p* = 0.071) were noted among SLE, other autoimmune diseases (OADs) and healthy controls. CXCL1, anti-dsDNA IgG, CRP, ESR, albumin, complement molecules C3 and C4, and neutrophil serum levels differed among the three study groups (*p* < 0.05). The positive rates of IgG ANA (χ^2^ = 46.159, *p* < 0.001) and HA IgG ANAs (χ^2^ = 16.856, *p* < 0.001) in SLE patients were significantly higher than those in OADs patients. Significant increases in CXCL1 serum levels were observed in the SLE group [1492.86 (735.47–2887.34) pg/ml] compared with the OADs group [174.23 (17.07–368.40) pg/ml] (*p* < 0.001) and healthy controls [13.58 (8.46–37.22) pg/ml] (*p* < 0.001). In addition, anti-dsDNA IgG [1093.37 (448.21–2215.30) U/L], ESR [25.45 (18.13–54.48) mm/h], CRP [1.66 (0.67–5.49) mg/mL] serum levels and the number of neutrophils [3.73(2.60–5.65) × 10^9^/mL] were significantly increased in the SLE group compared with healthy controls (*p* < 0.05). Furthermore, C3 [0.54 (0.34–0.77) g/L], C4 [0.09 (0.04–0.12) g/L] and albumin [37.65 (30.88–42.80) g/L] serum levels were lower in the SLE group compared with the OADs group and healthy controls (*p* < 0.05). Similarly, OADs patients had higher CXCL1, CRP and ESR serum levels compared with healthy controls (*p* < 0.001). However, no difference in CRP and ESR serum levels was noted between SLE group and OADs patients (*p* > 0.05). (Table 1).

**Table 1:** Characteristics of study participation

	SLE	OADs	Healthy controls	*p* value
	(n = 90)	(n = 56)	(n = 100)	
Sex (females/males)	83/7	44/12	87/13	0.058^a^
Positive IgG ANAs (%)	100% (90/90)	57% (32/56)	3% (3/100)	< 0.001^a^
Positive HA IgG ANA (%)	71% (64/90)	30% (17/56)	0	< 0.001^a^
	P_50_ (P_25_-P_75_)	P_50_ (P_25_-P_75_)	P_50_ (P_25_-P_75_)	
Age (years)	34 (27–45)	37(31–46)	33 (28–44)	0.071^a^
SLEDAI score	14 (10–18)	ND	ND	
CXCL1 (pg/mL)^c^	1492.86 (735.47–2887.34)	174.23 (17.07–368.40)	13.58 (8.46–37.22)	< 0.001^b^
An-dsDNA IgG (IU/L)^d^	1093.37 (448.21–2215.30)	10 (10–10)	10 (10–10)	< 0.001^b^
C3 (g/L)^e^	0.54 (0.34–0.77)	1.04 (0.86–1.27)	1.09 (0.96–1.23)	< 0.001^b^
C4 (g/L)^f^	0.09 (0.04–0.12)	0.21 (0.15–0.30)	0.255 (0.21–0.30)	< 0.001^b^
ESR (mm/h)^g^	25.45 (18.13–54.48)	19.5 (10.7–37.3)	12.75 (7.7–21.43)	< 0.001^b^
CRP (mg/mL)^h^	1.66 (0.67–5.49)	2.31 (0.92–8.20)	0.44 (0.30–0.57)	< 0.001^b^
Albumin (g/L)^i^	37.65 (30.88–42.80)	41.3 (35.55–44.53)	45.0 (43.30–46.60)	< 0.001^b^
Neu (×10^9^)^j^	3.73 (2.60–5.65)	3.29 (2.47–5.03)	3.23 (2.55–4.0)	0.030^b^

Characteristics of the study populations were shown in Table 1. SLE: Systemic lupus erythematosus; OADs: other autoimmune diseases; HAIgGANA: high-avidity antinuclear antibody (ANA) of the IgG isotype; ND: not detected. SLEDAI: SLE Disease Activity Index; C3/C4: Complement 3/4; ESR: erythrocyte sedimentation ratio; CRP: C-reactive protein; Neu: neutrophils

^a^Data was compared to different groups, the difference was statistically significant. The chi-squared test or Fisher's exact test were used

^b^Data was compared to different groups, the difference was statistically significant. The Kruskal–Wallis H test were used

^C^SLE versus healthy controls, *p* < 0.001; SLE versus OAD, *p* < 0.001; OAD versus healthy controls, *p* < 0.001

^d^SLE versus healthy controls, *p* < 0.001; SLE versus OAD, *p* < 0.001; OAD versus healthy controls, *p* = 0.002

^e^SLE versus healthy controls, *p* < 0.001; SLE versus OAD, *p* < 0.001; OAD versus healthy controls, *p* = 0.011

^f^SLE versus healthy controls, *p* < 0.001; SLE versus OAD, *p* < 0.001; OAD versus healthy controls, *p* = 0.007

^g^SLE versus healthy controls, *p* < 0.001; SLE versus OAD, *p* = 0.158; OAD versus healthy controls, *p* < 0.001

^h^SLE versus healthy controls, *p* < 0.001; SLE versus OAD, *p* = 0.094; OAD versus healthy controls, *p* < 0.001

^i^SLE versus healthy controls, *p* < 0.001; SLE versus OAD, *p* = 0.005; OAD versus healthy controls, *p* < 0.001

^j^SLE versus healthy controls, *p* = 0.007; SLE versus OAD, *p* = 0.352; OAD versus healthy controls, *p* = 0.242

### Comparisons of serum CXCL1 concentrations and anti-dsDNA IgG levels before and after treatment in SLE group

**Fig. 1:**
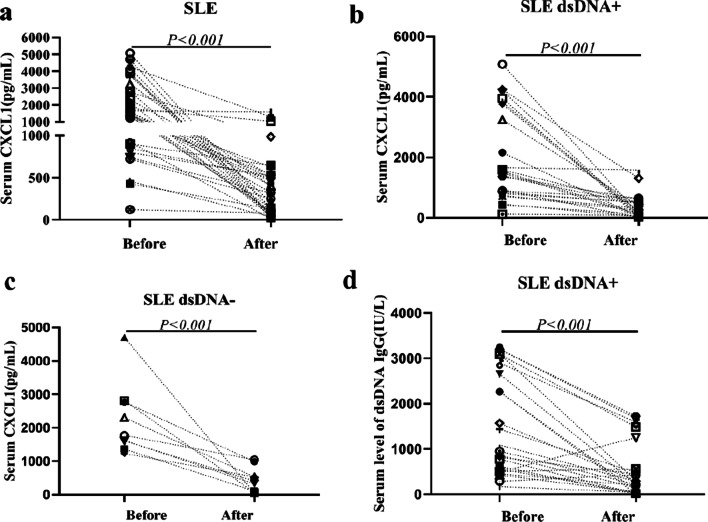
Comparisons of serum CXCL1 concentrations before and after treatment in SLE group, SLE anti-dsDNA + group and SLE anti-dsDNA- group (**a**–**c**). Comparison of serum anti-dsDNA levels before and after treatment in SLE anti-dsDNA + group (**d**)

In our study, the serum CXCL1 levels were measured before and after one year treatment of 32 patients in active SLE group. Then we subdivided the SLE patients into two groups, 23 patents in the SLE anti-dsDNA-positive group and 9 patients in the SLE anti-dsDNA-negative group before treatment as previous report [4]. Significant increases in serum CXCL1 levels were shown in the SLE group (*p* < 0.001) (Fig. 1a). Likewise, the same tendency towards a significant decrease in serum CXCL1 and anti-dsDNA levels after treatment in both subgroups (*p* < 0.001) (Fig. 1b–d).

Furthermore, we investigate whether the levels of CXCL1 were affected in 32 patients with SLE group with different treatments. The results indicated that the combined prednisone with hydroxychloroquine (HCQ) (65.6%, 21/32) was mainly treated. Second, a subset combined prednisone and HCQ and MMF plus tacrolimus (12.5%, 4/32) was treated as follows. Third, both prednisone and HCQ plus cyclophosphamide (CYC) and combined prednisone and HCQ with mycophenolate mofel (MMF) (9.4%, 3/32) were the same. Last, there was only one patient treated with combined prednisone with HCQ plus tacrolimus (3.1%, 1/32). However, we observed that there was no difference in CXCL1 levels decrease between different drug treatments after one year (*p* > 0.05).

### Relationship between serum CXCL1 concentrations in SLE patients with different disease activities, LN/non-LN patients, active/inactive LN patients, and high-avidity/low-avidity IgG ANA patients

**Fig. 2:**
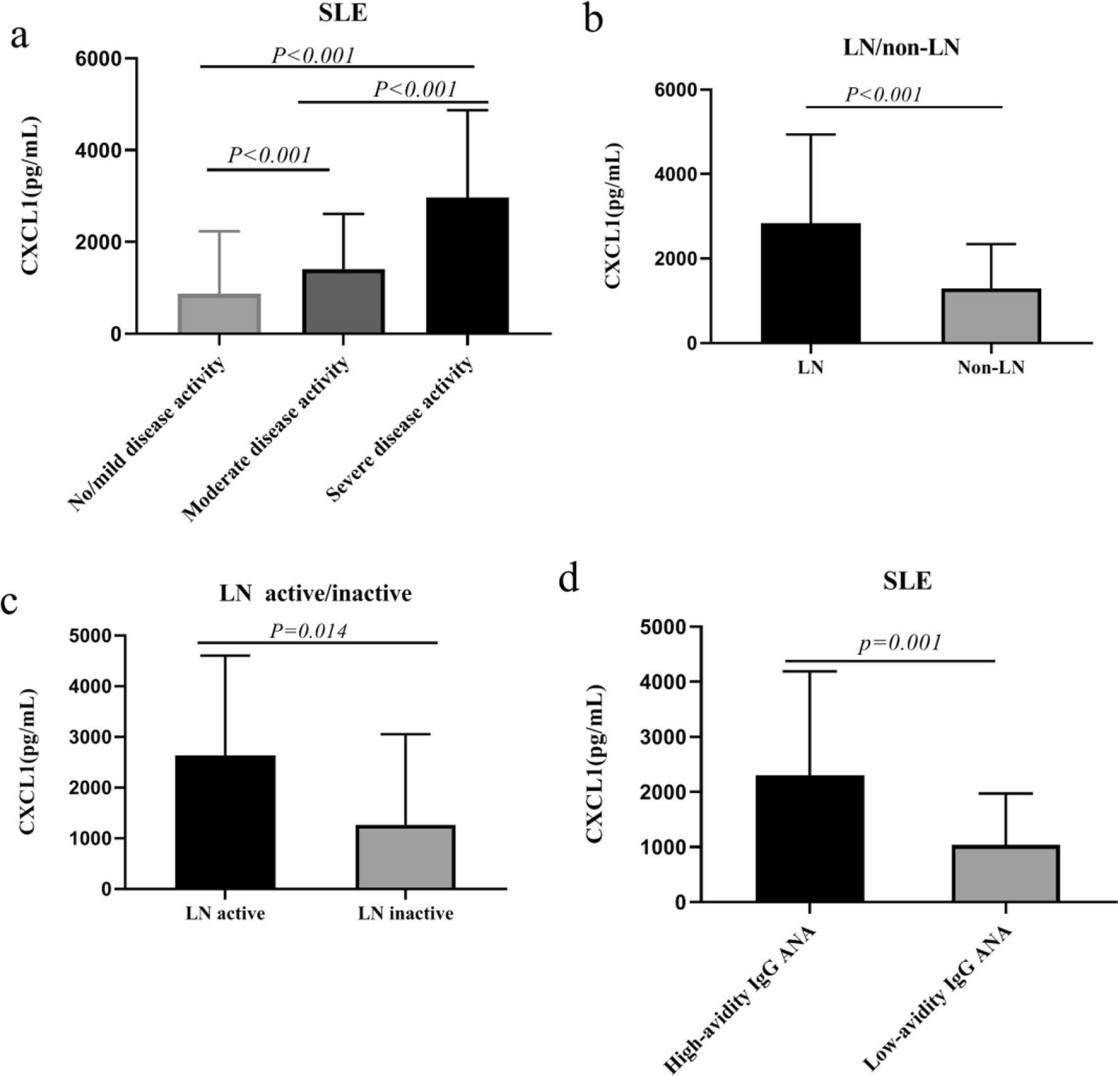
Relationship between serum concentration of CXCL1 in different disease activities, LN/non-LN cases, active/inactive LN cases, and high-avidity/low-avidity IgG ANA cases in SLE patients. LN: lupus nephritis

According to our previous report, we classified SLE patients into three groups based on disease activity [4]. Fifteen patients (17%, 15/90) were diagnosed with no/mild SLE disease activity (0 ≤ SLEDAI ≤ 9), 36 patients (40%, 36/90) were diagnosed with moderate SLE disease activity (10 ≤ SLEDAI ≤ 14), and 39 patients (43%, 39/90) were diagnosed with severe SLE disease activity (SLEDAI ≥ 15). Forty-one patients (46%, 41/90) were diagnosed with LN, and 68 (76%, 68/90) patients were diagnosed with high-avidity IgG ANAs of SLE. Significantly increased serum CXCL1 levels were noted in SLE patients with severe disease activity [2482.22 (1589.93–3760.29) pg/ml] compared with the no/mild disease activity [279.02 (122.38–1144.32) pg/ml] and moderate disease activity SLE [1066.96 (615.38–1618.66) pg/ml] groups (*p* < 0.001). (Fig. 2a). A significant increase in CXCL1 serum levels was observed in the LN group [2482.22 (1283.33–3900.12) pg/ml] and active LN group [2149.37 (1251.68–3818.64) pg/ml] compared with the non-LN group [1228.50 (400.46–1720.17) pg/ml] (*p* < 0.001) and inactive LN group [427.72 (186.17–2761.36) pg/ml] (*p* = 0.014) (Fig. 2b, c). Moreover, serum CXCL1 levels in the high-avidity IgG ANA group [1600.08 (937.31–3445.17) pg/ml] were significantly greater than those in the low-avidity IgG ANA group [803 (230.14–1691.90) pg/ml] (*p* = 0.001) (Fig. 2d).

### Correlation analysis of CXCL1 serum levels with laboratory parameters in study participants and factors that influence CXCL1 levels as assessed by multiple linear stepwise regression analysis

**Fig. 3:**
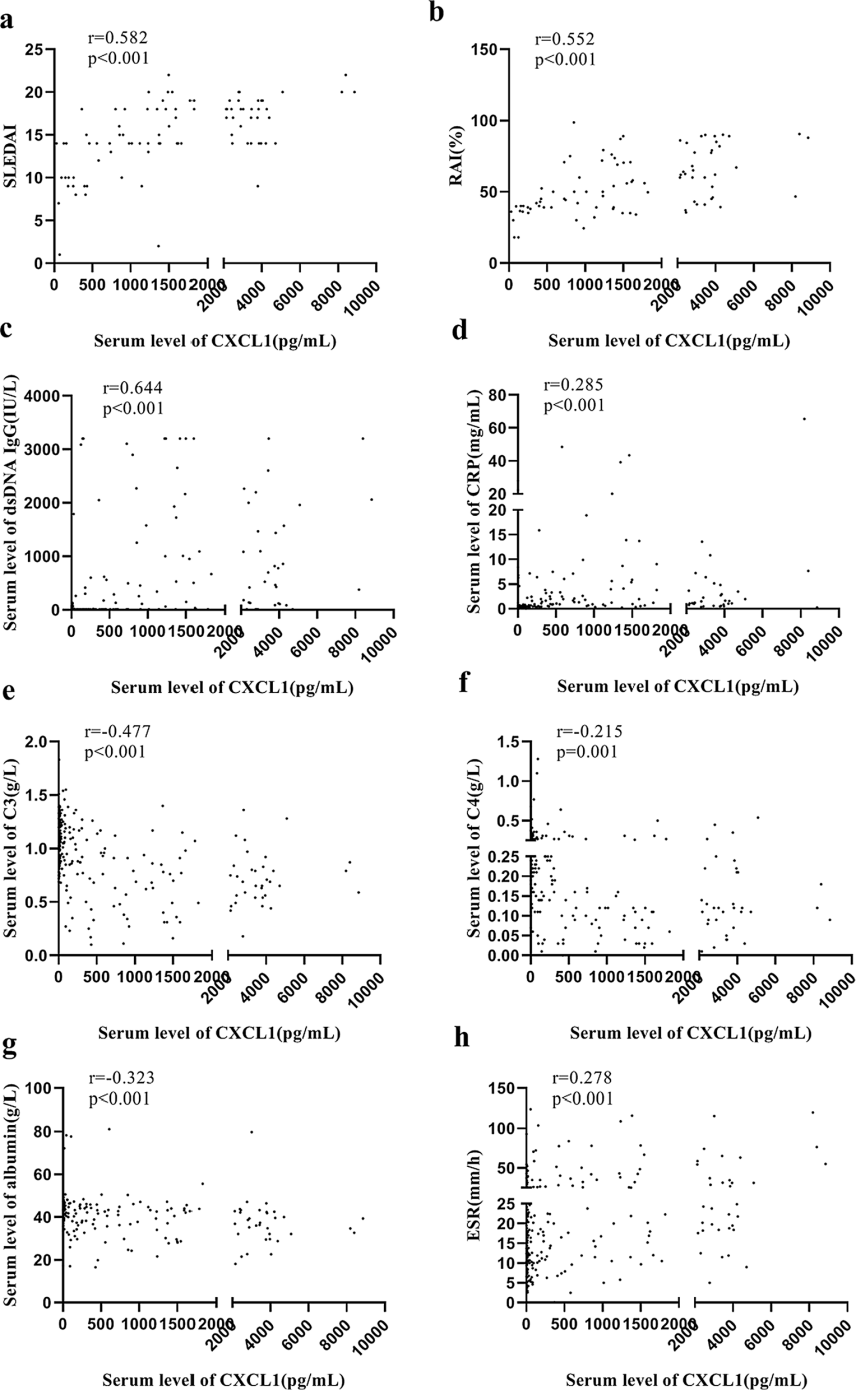
Correlation analysis of the serum levels of CXCL1 with SLEDAI, RAI of HA ANA IgG, serum levels of anti-dsDNA IgG, CRP, complements C3, C4,albumin and ESR in SLE patients. SLEDAI:SLE Disease Activity Index; RAI of HA ANA IgG, relative avidity indexof high-avidity IgG ANAs; C3/C4: complement3/4; ESR: erythrocyte sedimentation ratio; CRP: C-reactive protein; Neu: neutrophils

In our study, correlations were noted between serum levels of CXCL1 and other laboratory parameters. Serum CXCL1 concentrations in SLE patients were significantly correlated with the SLEDAI score and RAI of HA IgG ANAs (r = 0.582, *p* < 0.001; r = 0.552, *p* < 0.001; respectively) (Fig. 3a, b). Moreover, CXCL1 concentrations were positively related to anti-dsDNA IgG, CRP and ESR serum levels (r = 0.644, *p* < 0.001; r = 0.285, *p* < 0.001; r = 0.278, *p* < 0.001; respectively) (Fig. 3c, d, h). However, CXCL1 concentrations were negatively associated with C3, C4 and albumin serum levels (r =  − 0.477, *p* < 0.001; r =  − 0.215, *p* = 0.001; r =  − 0.323, *p* < 0.001; respectively) (Fig. 3e–g).

To further verify laboratory parameters influencing CXCL1 levels, we performed multiple linear stepwise regression analysis. The independent variables included the presence of IgG ANA, the presence of HA; the levels of anti-dsDNA IgG, CRP, ESR, albumin, and C3/C4; and the number of neutrophils. CXCL1 levels served as the dependent variable in the statistical analysis. The results showed that the presence of IgG ANA (*p* < 0.001), the presence of high-avidity IgG ANA (*p* = 0.001) and the logarithmic level of anti-dsDNA IgG (*p* = 0.021) were significantly associated with the logarithmic level of CXCL1. The standard partial regression coefficients (95% CI) were 2.371 (1.734–3.009), 1.231 (0.520–1.937) and 0.409 (0.062–0.755) (Table 2).

**Table 2:** Influencing factors on CXCL1 by multiple linear stepwise regression analysis

Confounding factor	Lg CXCL1		
	β Coefficient (95% CI)	*p* value	Adjust R^2^
Positive IgG ANA	2.371 (1.734–3.009)	< 0.001	
Positive HA ANA IgG	1.231 (0.525–1.937)	0.001	0.604
Lg anti-dsDNA	0.409 (0.062–0.755)	0.021	

HA ANA IgG, high-avidity IgG ANAs; Lg CXCL1/anti-dsDNA represent the logarithmic level of CXCL1 and anti-dsDNA

### CXCL1 potentially represents a diagnostic marker to distinguish active SLE from inactive SLE and LN from non-LN

**Fig. 4:**
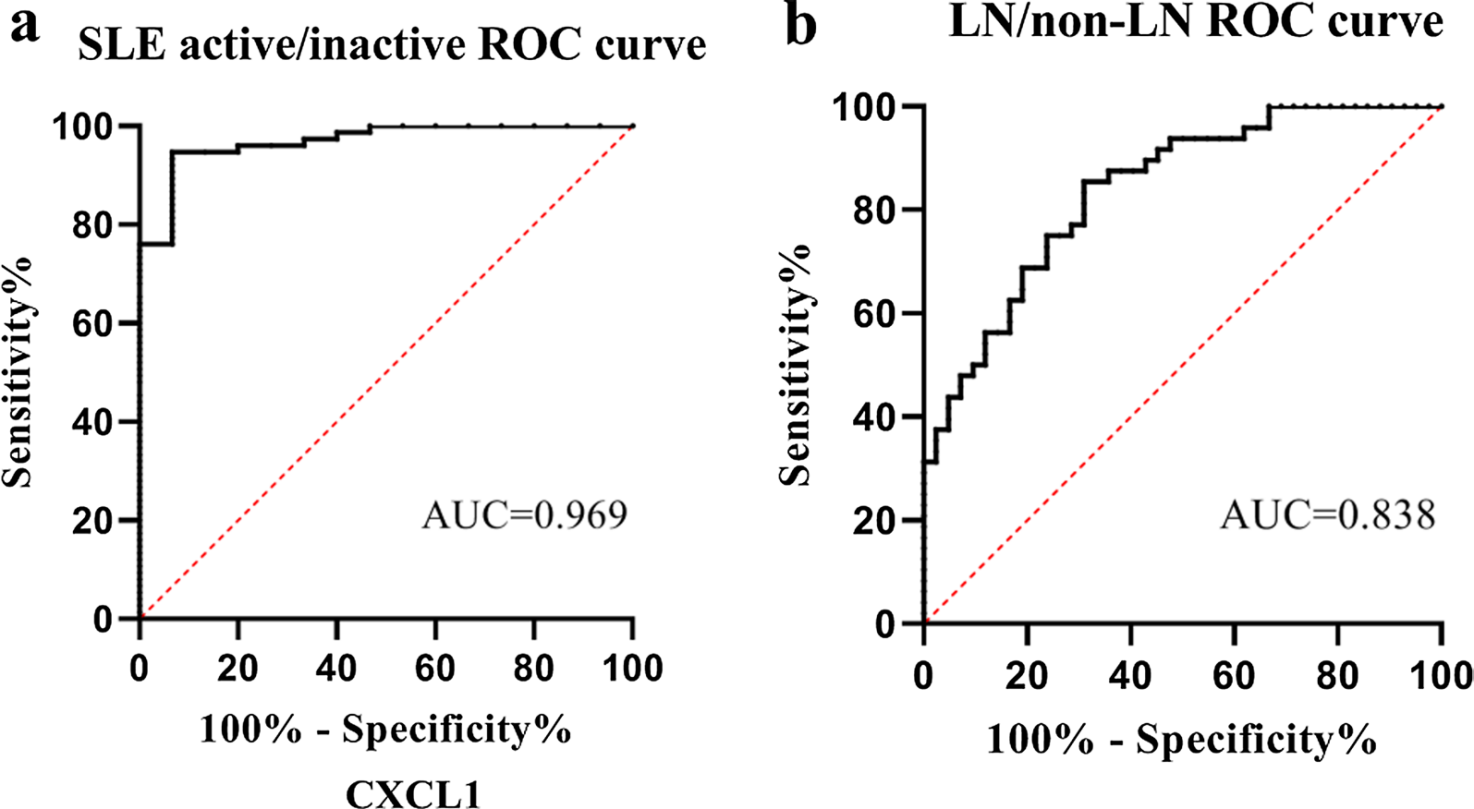
ROC curves analysis using CXCL1 for distinguishing active SLE from inactive SLE and LN from non-LN in SLE patients

In our study, we estimated the diagnostic potential and accuracy of serum CXCL1 levels for both active SLE and LN based on ROC curves (Fig. 4). Serum CXCL1 levels had a strong role in differentiating active SLE (SLEDAI > 10) from inactive SLE (SLEDAI < 10) and non-LN patients with AUCs of 0.969 (95% CI 0.933–1.0, *p* < 0.0001) and 0.838 (95% CI 0.758–0.918, *p* < 0.0001), respectively. The cutoff values to distinguish between active SLE and inactive SLE as well as LN and non-LN were 1182.17 pg/mL and 1500.31 pg/mL. The sensitivity and specificity values were 76% and 100%, and 76% and 75%, respectively.

## Discussion

For the first time, we demonstrate that serum CXCL1 levels were significantly higher in patients with SLE than in patients with other autoimmune diseases and healthy controls. Moreover, CXCL1 serum concentrations were markedly increased in the active SLE and LN groups. Furthermore, circulating CXCL1 levels were correlated with the SLE disease activity (SLEDAI) score, RAI of HA IgG ANAs, serum anti-dsDNA IgG levels, and other laboratory parameters. In addition, CXCL1 potentially represents a diagnostic marker to distinguish active SLE and LN given its high sensitivity and specificity.

To date, circulating CXCL1 has never been evaluated as a marker of disease activity in patients with SLE despite a broad theoretical basis in the literature suggesting that chemokines contribute to the pathogenesis of SLE and LN. It has been shown that serum CXCL1 concentrations are specific to systemic sclerosis (SSc) and correlated with the involvement of internal organs, especially pulmonary damage [17]. Similar findings were presented by Lisi et al. [18], who reported significantly higher CXCL1 levels in Sjögren's syndrome (SS) tissues than in healthy controls. Although there are few reports that support a possible role of CXCL1 during inflammation and neovascularization in autoimmune diseases, we examined CXCL1 in SLE serum in this study. The results showed that serum CXCL1 levels were significantly increased in patients with SLE compared with patients with other autoimmune diseases, including SS and SSc. However, Furuse et al. [17] failed to detect higher serum CXCL1 levels in SLE patients. In addition to the difference between patients assessed, these discrepant results may be due to the small sample numbers analyzed or low sensitivity of the ELISA system used. This report evaluated only 15 patients with SLE. Moreover, we further assessed the regulatory response of serum CXCL1 levels and anti-dsDNA IgG levels in active SLE patients after treatment. The results were shown that CXCL1 had important prognostic stool in SLE, especially in the absence of anti-dsDNA IgG antibodies. Therefore, CXCL1 might be useful tools for monitoring the extent of inflammation in SLE patients. In addition, CXCL1 serum levels were particularly increased in LN compared with non-LN patients. It is hypothesized that CXCL1 may be upregulated in conjunction with immune complex deposition and occurs prior to cellular infiltration, proteinuria and kidney damage by binding to the CXCR2 receptor in mesangial cells or glomerular cells. This notion is consistent with the previously reported that chemokines initiated leukocyte infiltration and precede proteinuria and renal damage in lupus nephritis in MRL/lpr mice [6].

To identify factors mediating CXCL1 levels, we examined the association with CXCL1 and markers of disease activity in SLE. Consistent with previous reports on CXCL13 [4], CXCL1 serum concentrations were associated with the SLEDAI, RAI of HA IgG ANAs, and serum levels of anti-dsDNA IgG in the present study. Of interest, the presence of IgG ANAs, HA IgG ANAs and anti-dsDNA IgG influence CXCL1 levels in SLE patients. First, it is suggested that the peak concentration of the secreted chemokine CXCL1 contributes to the stimulation of a large number of autoantibodies in SLE patients in an active disease state, and chemokine dysregulation in SLE have the potential for identifying patient subsets before the onset of clinical disease and during established disease [8]. Second, it is believed that chemokine CXCL1 may be involved in the pathogenesis of LN by coordinating the pro-inflammatory microenvironments, recruiting immune cell subsets into the kidney and inducing local activation of immune effector cells, resulting in significant tissue damage [7, 10]. Third, it has been proposed that after chemokines are produced by resident cells, infiltrating leukocytes become the source of chemokine production, leading to amplification loops [7, 19].

The previously introduced “HA IgG ANA” can be used to distinguish early-stage SLE from SLE that has been active for some time [1]. Our data showed increased CXCL1 serum concentrations in the HA group compared with the active SLE group and LN group. Furthermore, we also discovered that CXCL13 serum levels were positively related to the RAI of HA IgG ANAs. In summary, our results indicate that the chemokine CXCL1 is upregulated prior to the inflammatory process and responds better to early aggressive treatment, which is consistent with the role of CXCL13 in SLE reported in previous studies [4, 20]. Of note, other markers, including complement activation products (e.g., C3 and C4), erythrocyte sedimentation rate (ESR), and C-reactive protein (CRP), have been used as indirect serological markers of SLE that correlated well with disease activity [21]. Consistent with their role in the disease activity process of SLE, our results showed that CXCL1 serum levels were marginally related to these markers. Further support for this association is provided by other observations that elevated CXCL1 levels are associated with reduced serum albumin levels, which is related to increased albumin catabolism due to chronic inflammation and/or insufficient protein and caloric intake in patients with SLE [22]. These findings highlight that CXCL1 is a strong marker of increased disease activity and organ damage during the course of SLE. Additionally, continuous measurements may be useful in assessing disease activity and impairment in SLE patients with elevated CXCL1 levels during the disease course.

Although it is clear that chemokines play an important role in the large influx of leukocytes to the site of tissue injury, the synergistic role of cytokines, adhesion molecules, and other inflammatory mediators must be considered. For example, CD4+ T cell- and γδ T cell-derived IL-17F induces the expression and production of the chemokines CXCL1 and CXCL5, which subsequently attract CXCR2-expressing neutrophils to the inflamed kidney [23]. This phenomenon results in the destruction of normal kidney tissue and consequent loss of renal function in C57BL/6J gene knockout mice [24]. In addition, Brown et al. [25] reported that the effect of TLR4 on both circulating leukocytes and intrinsic renal cells contributes to the inflammatory effects of glomerular antibody deposition in TLR4-deficient mice, which depends on endogenous renal cells that at least partially produce CXCL1 and CXCL2 chemokines. These studies have highlighted the potential importance of role of CXCL1 in renal cells in autoimmune diseases. Interestingly, CXCL1 concentrations were increased in patients with LN and active disease in the present study. It is possible to hypothesize that the high CXCL1 serum levels may be due to overexpression in kidneys. However, peripheral blood neutrophils may represent another source of circulating CXCL1, and this notion is consistent with the increased levels of blood neutrophils in SLE patients in our study.

Our study has several limitations. First, the experiment included only a limited number of enrolled participants. Therefore, more study participants are needed to further validate our proposed finding. In addition, due to the retrospective nature of this study, we did not directly assess our hypothesis that the mechanism of disease activity affects the production of CXCL1 in vitro. We will continue to examine CXCL1 levels in kidney tissues collected from LN patients and to assess the role of CXCL1 serum levels in a mouse model.

In conclusion, our study provides a clinical evaluation of the CXCL1 serum levels in SLE patients. CXCL1 serum concentrations could be used to differentiate disease activity between SLE and LN. Furthermore, the co-occurrence of IgG ANA and HA IgG ANAs may be associated with the CXCL1 levels in patients with SLE. Therefore, the chemokine CXCL1 may be implicated in the pathogenesis/inflammatory process in lupus.

## Data Availability

The datasets generated and analysed during the current study are not publicly available due to limitations of ethical approval involving the patient data and anonymity but are available from the corresponding author on reasonable request.
